# An abnormal metabolism-related gene, ALG3, is a potential diagnostic and prognostic biomarker for lung adenocarcinoma

**DOI:** 10.1097/MD.0000000000038746

**Published:** 2024-09-13

**Authors:** Abdusemer Reyimu, Xiang Cheng, Wen Liu, Aihemaitijiang Kaisaier, Xinying Wang, Yinzhong Sha, Ruijie Guo, Pawuziye Paerhati, Maimaituxun Maimaiti, Chuanjiang He, Li Li, Xiaoguang Zou, Aimin Xu

**Affiliations:** a Department of Laboratory Medicine, The First People’s Hospital of Kashi, Kashi City, China; b The First People’s Hospital of Kashi, Kashi City, China.

**Keywords:** ceRNA, immune infiltration, lung adenocarcinoma (LUAD), prognosis, risk model

## Abstract

**Background:**

To explore the abnormal metabolism-related genes that affect the prognosis of patients with lung adenocarcinoma (LUAD), and analyze the relationship with immune infiltration and competing endogenous RNA (ceRNA) network.

**Methods:**

Transcriptome data of LUAD were downloaded from the Cancer Genome Atlas database. Abnormal metabolism-related differentially expressed genes in LUAD were screened by the R language. Cox analysis was used to construct LUAD prognostic risk model. Kaplan–Meier test, ROC curve and nomograms were used to evaluate the predictive ability of metabolic related gene prognostic model. CIBERSORT algorithm was used to analyze the relationship between risk score and immune infiltration. The starBase database constructed a regulatory network consistent with the ceRNA hypothesis. IHC experiments were performed to verify the differential expression of ALG3 in LUAD and paracancerous samples.

**Results:**

In this study, 42 abnormal metabolism-related differential genes were screened. After survival analysis, the final 5 metabolism-related genes were used as the construction of prognosis model, including ALG3, COL7A1, KL, MST1, and SLC52A1. In the model, the survival rate of LUAD patients in the high-risk subgroup was lower than that in the low-risk group. In addition, the risk score of the constructed LUAD prognostic model can be used as an independent prognostic factor for patients. According to the analysis of CIBERSORT algorithm, the risk score is related to the infiltration of multiple immune cells. The potential ceRNA network of model genes in LUAD was constructed through the starBase database. IHC experiments revealed that ALG3 expression was upregulated in LUAD.

**Conclusion:**

The prognostic model of LUAD reveals the relationship between metabolism and prognosis of LUAD, and provides a novel perspective for diagnosis and research of LUAD.

## 1. Introduction

Lung cancer is a malignant tumor originating from the bronchial mucosa or glands, among which lung adenocarcinoma (LUAD) is 1 of its important tissue subtypes, with incidence and mortality at a high level.^[1]^ However, the etiology has not yet been fully clarified, and most of them are believed to be closely related to atmospheric pollution, hereditary factors, and smoking.^[2]^ Heterogeneity of LUAD poses a challenge for predicting the prognosis of patients. Clinical manifestations, morphology, molecular features, therapeutic effects and prognosis vary widely from case to case. Clinically, tumor stage, histological grading, and molecular subtype are commonly used as prognostic factors for LUAD. However, the ability of clinical features to predict LUAD prognosis is limited. An inaccurate grasp of the prognostic risk of LUAD patients may lead to overtreatment of low-risk patients and inappropriate treatment of high-risk patients.^[3]^ Therefore, it is of critical importance to construct a reliable and clinician-friendly prognostic model of lung adenocarcinoma for targeted interventions.

Metabolic changes are 1 of the hallmarks of tumor cells, and it is generally believed that this is achieved through increased aerobic fermentation.^[4]^ Metabolic reprogramming represents cancer-related metabolic changes during tumorigenesis, and therapeutic approaches targeting this mechanism of tumor metabolic reprogramming are currently being explored.^[5]^ Within tumor cells, many metabolic changes are generated to meet the energy and synthetic requirements of tumors, which has become an important feature of tumors.^[6]^ In this study, a model combining multiple cancer metabolism-related genes was constructed to predict the prognostic risk of LUAD patients. Through differential analysis of metabolism-related genes in the LUAD Cancer Genome Atlas Program (TCGA) database, metabolism-related differential genes were screened, and a prognostic model was constructed through Cox regression to find therapeutic targets, predict the prognosis of LUAD patients, and formulate an individualized diagnostic and therapeutic plan.

## 2. Materials and methods

### 2.1. Material

LUAD transcription data involved in this research were from TCGA database, including 515 LUAD and 59 normal samples. The GSE50081 data set was downloaded from the Gene Expression Omnibus database (GEO, https://www.ncbi.nlm.nih.gov/geo/). The GPL570 (Affymetrix Human Genome U133 Plus 2.0 Array) was used as the platform information, including 127 lung adenocarcinoma samples with prognostic information. Abnormal metabolism-related genes (153 in total) were downloaded from MSigDB database in Gene Set Enrichment Analysis web page (Supplementary data S1, Supplemental Digital Content, http://links.lww.com/MD/N316).

### 2.2. Acquisition of DEGs related to metabolism

After normalizing transcription data with the “edgeR” package,^[7]^ the differentially expressed genes (DEGs) in LUAD were screened. False discovery rate (FDR) < 0. 05 and |log2 fold change| >1 were considered to be significantly differentially expressed. Wayne map was used to extract genes related to abnormal metabolism in DEGs. In this study, the STRING database was utilized to explore the interactions between differentially expressed abnormal metabolism-related genes.

### 2.3. Functional enrichment analysis

The “clusterprofiler” package^[8]^ was used for GO function enrichment and KEGG pathway analysis. The “goplot” package is used to calculate *z*-score. The significantly enriched GO and KEGG results are displayed by chord graph.

### 2.4. Construction of prognostic risk model

“Survival” package is used to screen abnormal metabolic genes related to prognosis in LUAD. The prognostic related metabolic genes were included in the multivariate analysis to obtain the LUAD prognostic risk model. The patient risk score was calculated according to the multiplication of gene coefficient and gene expression in LUAD model. According to the median of the risk score, the LUAD patients were divided into high-risk group and the low-risk group. Kaplan–Meier survival method was used to analyze the survival of high-risk group and the low-risk group, and ROC curve and area under curve (AUC) were used to evaluate the accuracy of the prognostic risk model. The nomogram was drawn by using risk score and clinical information.

### 2.5. Immune cell infiltration

CIBERSORT method was used to calculate the proportion of 22 kinds of immune cells. Pearson test was used to analyze the correlation between risk score and immune infiltration, and box plot and scatter plot were used to show the differences between different risk subgroups. Pearson correlation test was used to analyze the expression correlation between model genes and immune checkpoint related genes in LUAD.

### 2.6. Validation of model gene expression

Through HPA database (https://www.proteinatlas.org/), we can analyze the expression of model genes at the protein level. Through CCLE database, we can analyze the expression of model genes in lung adenocarcinoma cell lines. Then, the genetic alteration of model genes in LUAD was explored through cBioPortal database (https://www.cbioportal.org/).

### 2.7. Prediction of ceRNA network construction

The target miRNA of model gene mRNA was predicted by starBase database and analyzed by 7 prediction programs (PITA, miRanda, DIANA microT, PicTar, miRmap, RNA22, and TargetScan). Target miRNA screening conditions: programNum ≥ 1, CLIP-Data ≥ 1, pan-Cancer ≥ 1, Degradome-Data ≥ 1. In addition, we further analyzed the correlation between target miRNA and mRNA, and screened miRNAs that were more suitable for ceRNA hypothesis. Based on the miRNA screened by the above methods, upstream lncRNA of miRNA was predicted. The correlation between miRNA and lncRNA was further analyzed in order to screen lncRNA more suitable for ceRNA conditions. By comprehensively analyzing “miRNA-mRNA” and “miRNA-lncRNA,” the “lncRNA-miRNA-mRNA” network of LUAD was established.

### 2.8. Immunohistochemistry (IHC) staining

Tissue microarrays were constructed by Shanghai Joly Bio-Tech Co. (Joly Bio-Tech Co., Ltd, Shanghai, China). The tissue array contained 1.5 mm diameter formalin-fixed paraffin-embedded tissue disks containing a total of 32 LUADs and 28 adjacent tissues. Tissue microarrays were serially excised and cut into 3 to 5 μM thick slices. First, xylene was added to deparaffinize, dehydrated with anhydrous ethanol, boiled in citrate buffer for 10 minutes to repair the antigen, and blocked with endogenous peroxidase. Then the primary antibody (anti-ALG3 antibody, diluted at 1:200) was added and incubated at 4 °C overnight. The secondary antibody was added and incubated at 25 °C for 1 hour, rinsed with PBS (Phosphate Buffered Saline), DAB (3,3′-diaminobenzidine) color development, hematoxylin counterstaining, gradient ethanol and xylene dehydration, and neutral resin sealing. IHC stained sections were independently analyzed and scored by an experienced pathologist. Percentage of positively stained cells and staining intensity were evaluated microscopically. Staining score: 0 to 1, negative; 1 to 2, weak; 2 to 3, moderate; 3 to 4, strong.

## 3. Results

### 3.1. Screening of abnormal metabolic genes

**Figure 1.**
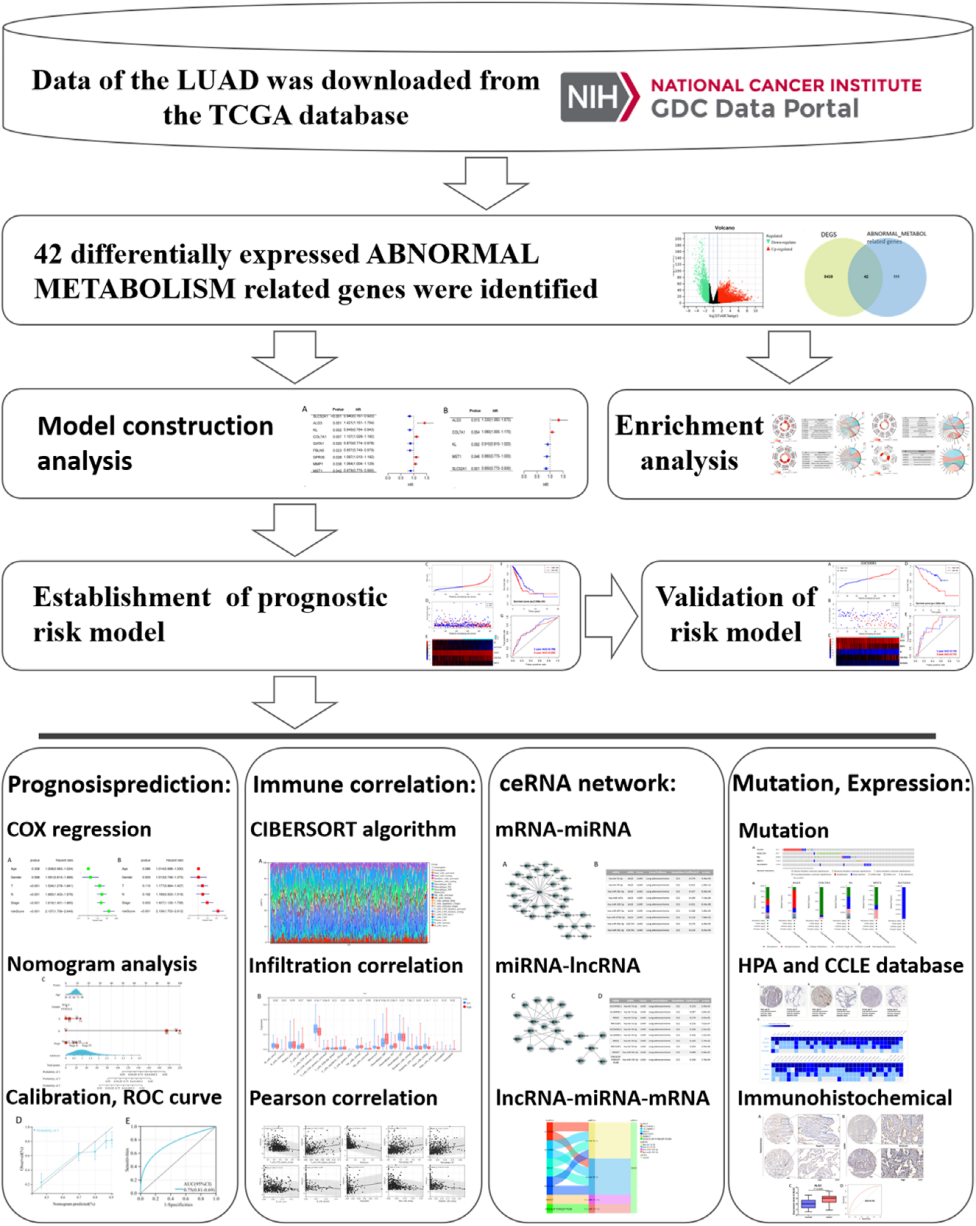
Research designs and process of this study.

**Figure 2.**
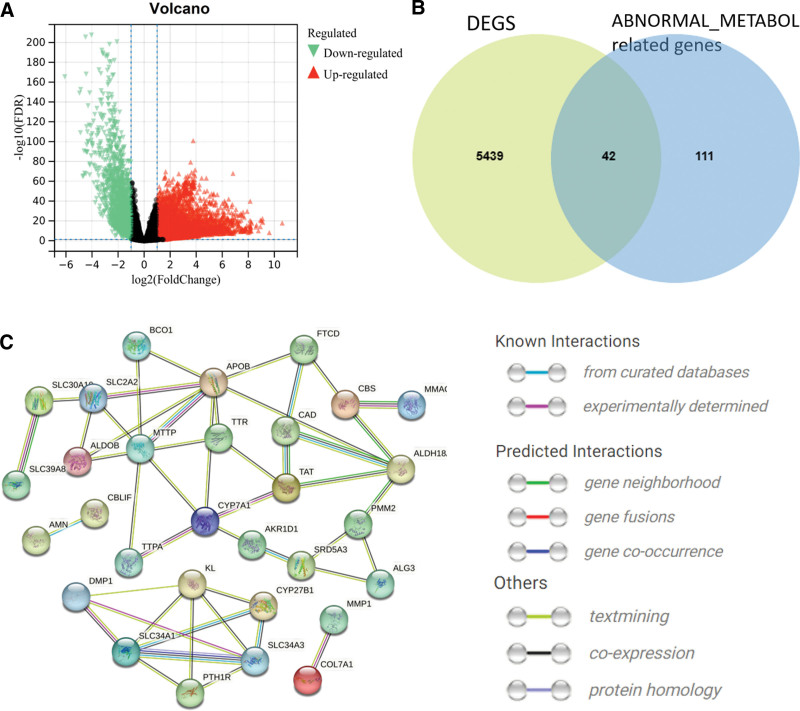
Screening for abnormal metabolism-related genes. (A) Volcano map of DEGs differentially expressed between LUAD and normal tissues. The selection criteria are |log2FC| >1, FDR value < 0.05 (Red dots indicate log2FC > 1 and FDR value < 0.05. Green dots indicate log2FC < 1 and FDR value < 0.05). (B) Extraction of differentially expressed genes related to abnormal metabolism. Among them, 42 metabolic related genes abnormally express. (C) Differentially expressed metabolic protein interaction network.

We first designed and described the research process (Fig. 1). After obtaining the gene expression amount of 515 LUAD cases and 59 normal lung tissues, the data were standardized, and then the differential expression analysis was performed on LUAD and normal tissues. A total of 5481 DEGs (*P* < .05) were screened out (Fig. 2A), of which 42 genes belonged to abnormal metabolism-related genes (Fig. 2B). The prediction of the interaction between metabolism-related genes is shown in the following figure (Fig. 2C).

### 3.2. Enrichment analysis

**Figure 3.**
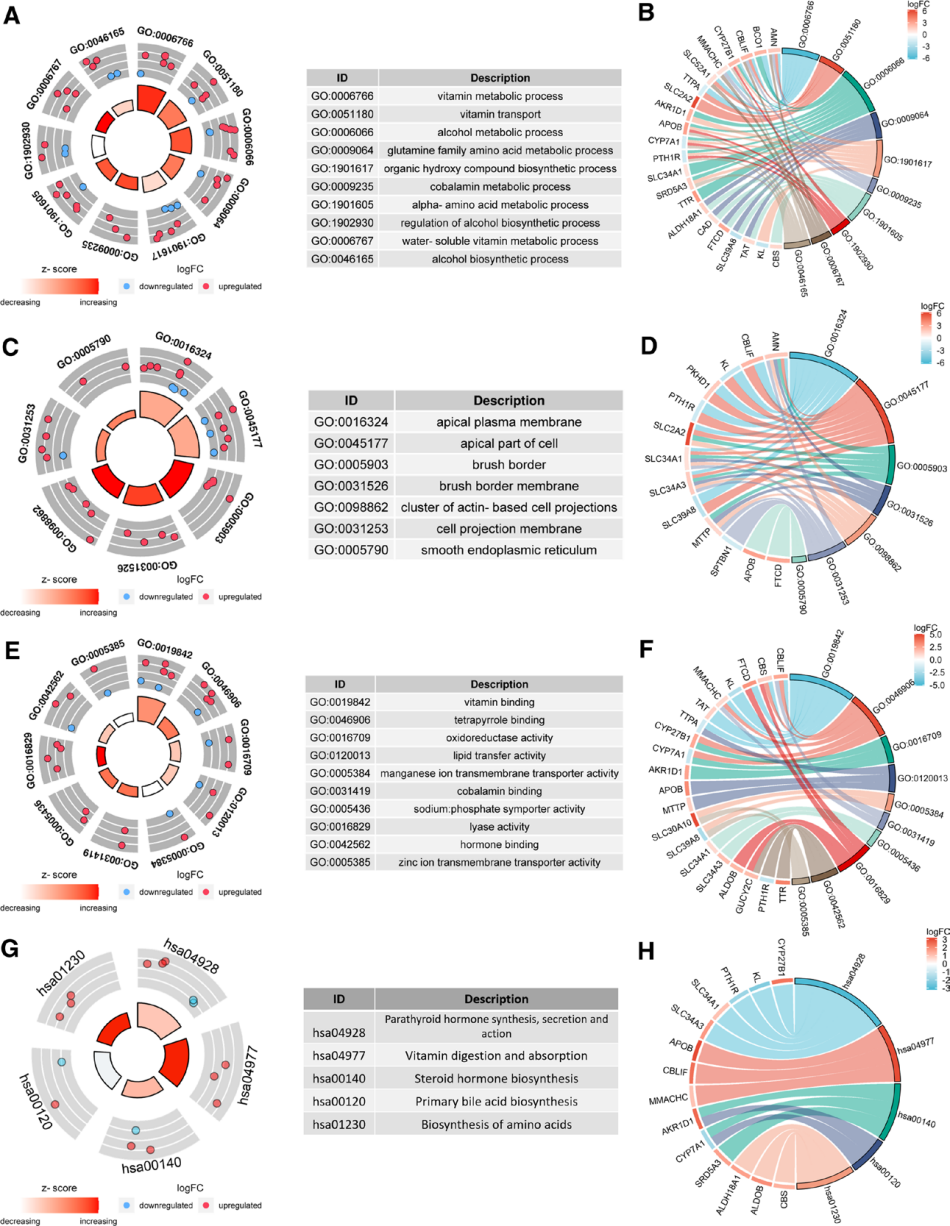
Functional enrichment analysis of 42 differentially expressed abnormal metabolism related genes. (A) Top 10 BP terms in GO enrichment analysis. The outer circle of the scatterplot shows the logFC identifier of the gene in each term. Red circles indicate up-regulated genes and blue circles indicate downregulated genes. (B) Distribution of genes corresponding to each term in BP. (C) Top 10 CC terms in GO enrichment analysis. (D) Distribution of genes corresponding to each term in CC. (E) Top 10 MF terms in GO enrichment analysis. (F) Distribution of genes corresponding to each term in MF. (G) Top 10 terms in the KEGG pathway. (H) Distribution of genes corresponding to each term in KEGG pathway.

The top ten biological processes (BP) in the GO functional enrichment results included vitamin metabolic process, vitamin transport, alcohol metabolic process, glutamine family amino acid metabolic process, organic hydroxy compound biosynthetic process, cobalamin metabolic process, alpha-amino acid metabolic process, regulation of alcohol biosynthetic process, water-soluble vitamin metabolic process and alcohol biosynthetic process (Fig. 3A). The chord plot shows the distribution of genes corresponding to BP (Fig. 3B). The top ten cellular components (CC) contain apical plasma membrane, apical part of cell, brush border, brush border membrane, cluster of actin-based cell projections, cell projection membrane and smooth endoplasmic reticulum (Fig. 3C). The chord plot shows the distribution of genes corresponding to CC (Fig. 3D). The top ten molecular functions (MF) include vitamin binding, tetrapyrrole binding, oxidoreductase activity, lipid transfer activity, manganese ion transmembrane transporter activity, cobalamin binding, sodium:phosphate symporter activity, lyase activity, hormone binding, zinc ion transmembrane transporter activity (Fig. 3E). The chord plot shows the distribution of genes corresponding to MF (Fig. 3F). The KEGG pathway focuses on parathyroid hormone synthesis, secretion and action, vitamin digestion and absorption, steroid hormone biosynthesis, primary bile acid biosynthesis, biosynthesis of amino acids (Fig. 3G). The chord plot shows the distribution of genes corresponding to the KEGG pathway (Fig. 3H).

### 3.3. Construction of prognostic risk model

**Figure 4.**
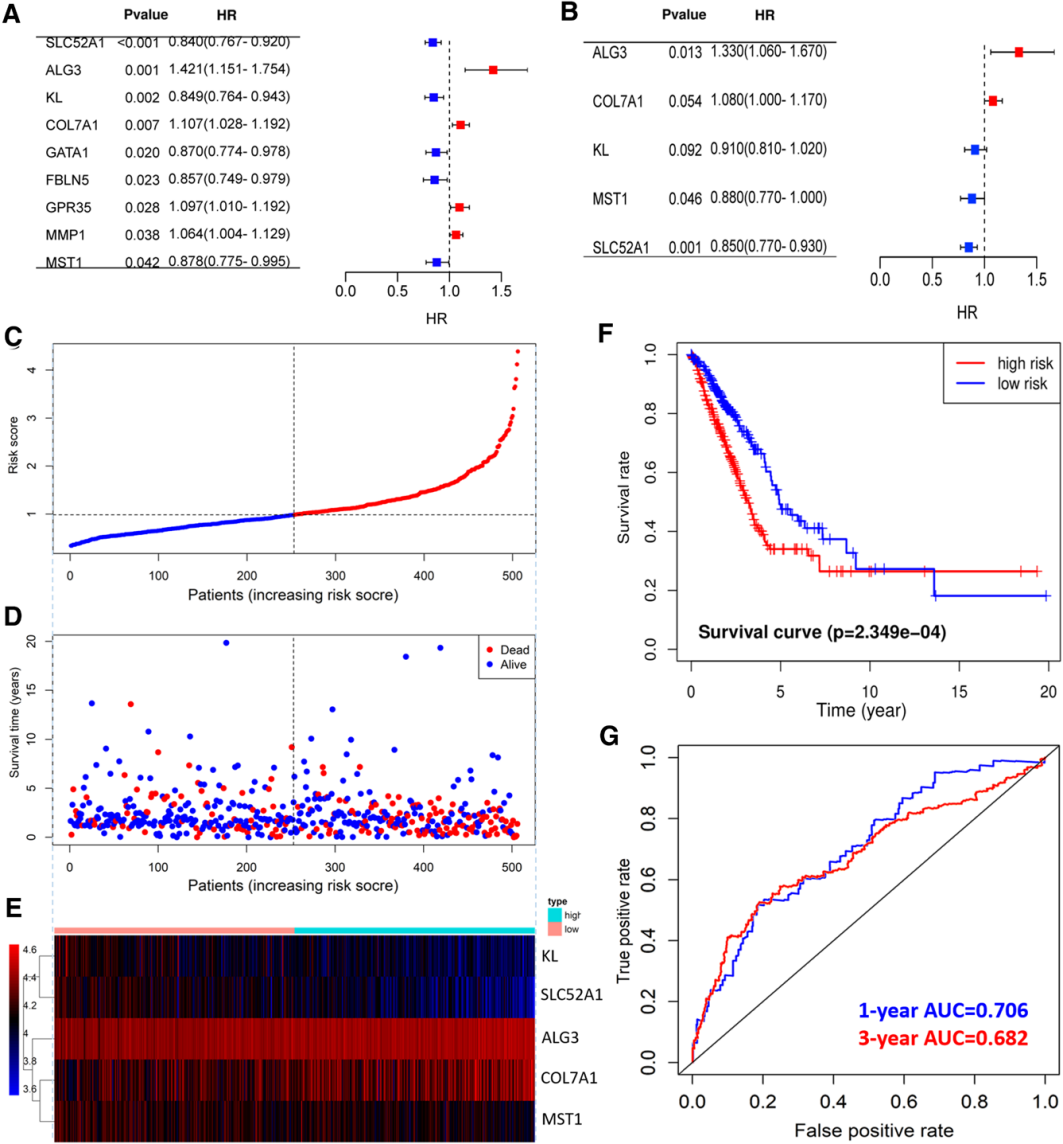
Establishment and evaluation of prognostic risk model. (A) Univariate Cox analysis of abnormal metabolism-related genes. (B) Multivariate Cox analysis of abnormal metabolism related genes. (C) Scatter plot of LUAD patient risk scores from low to high. Red dots represent high-risk groups. Blue dots represent the low-risk group. (D) Scatter plot distribution of survival time and survival status corresponding to risk scores of high and low-risk groups. (E) Heat map of expression trend of 6 model genes in LUAD patients. The expression was gradually increased from blue to red. (F) Comparison of survival rate between high and low-risk groups. (G) ROC curves to assess the effectiveness of risk models in predicting 1 - and 3-year outcomes.

Univariate Cox analysis of abnormal metabolic genes was carried out with survival package, and 9 prognosis related genes were screened out (Fig. 4A). Multivariate Cox regression analysis was further included to establish a multigene prognosis risk model (Fig. 4B). The risk value of each patient was calculated based on the selected prognostic gene expression multiplied by the sum of multivariate Cox regression coefficients. LUAD patients were sorted by risk score and divided into high-risk subgroup and low-risk subgroup with the median value as the boundary (Fig. 4C). The distribution of survival status shows that the death population is denser in the high-risk group (Fig. 4D). The heat map shows the expression trend of the 5 model genes (Fig. 4E). The survival rate of LUAD patients in the high-risk group in the model was lower (Fig. 4F). The AUC values for the ROC curve assessment risk model to predict 1- and 3-year prognostic efficacy in LUAD patients were 0.706 and 0.682, respectively (Fig. 4G).

### 3.4. Validation of prognostic risk model in the GEO dataset

**Figure 5.**
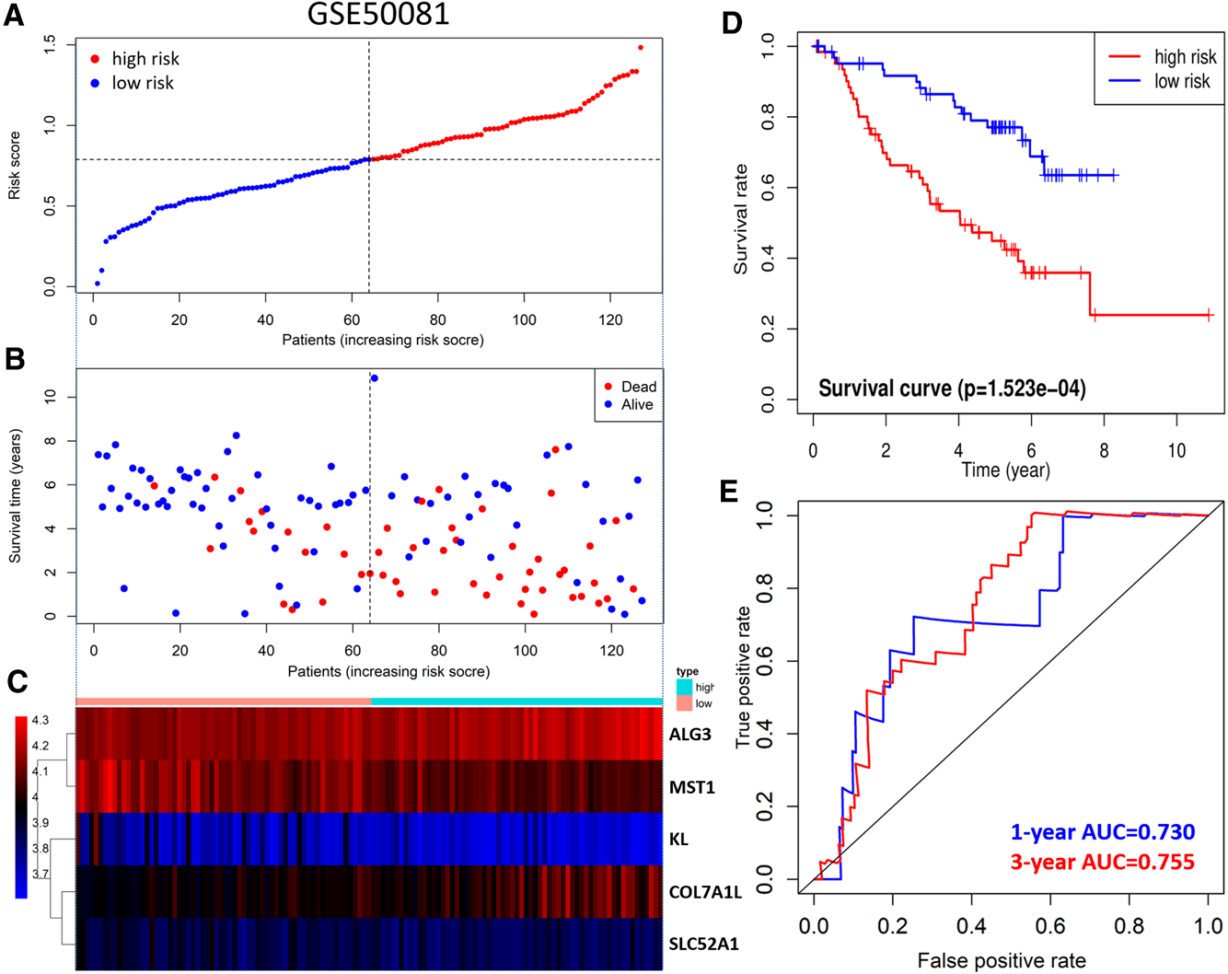
Validation of prognostic risk model in GEO database. (A) scatter plot of patient risk scores from low to high. Red dots represent high-risk groups. Blue dots represent the low-risk group. (B) scatter plot distribution of survival time and survival status. Red represents death cases and blue represents living cases. (C) Heat map of gene expression trends in 6 models. Expression is gradually increased from blue to red. (D) Kaplan–Meier survival curves for OS in high-risk and low-risk groups. (G) ROC curves to assess the effectiveness of risk models in predicting 1 - and 3-year outcomes.

Risk score of LUAD patients in the GSE50081 dataset was calculated according to the risk score formula. Patients were divided into high-risk subgroup and low-risk subgroup according to the median value (Fig. 5A). The distribution of survival status was similar to that of TCGA data, and death cases were more dense in the high-risk subgroup (Fig. 5B). The heat map shows the expression trend of the 6 model genes (Fig. 5C). The patients in the high-risk subgroup of the model had a poor prognosis (Fig. 5D). The AUC values for the ROC curve assessment risk model to predict 1- and 3-year prognostic efficacy in LUAD patients were 0.730 and 0.755, respectively (Fig. 5E).

### 3.5. Prognostic value of risk model

**Figure 6.**
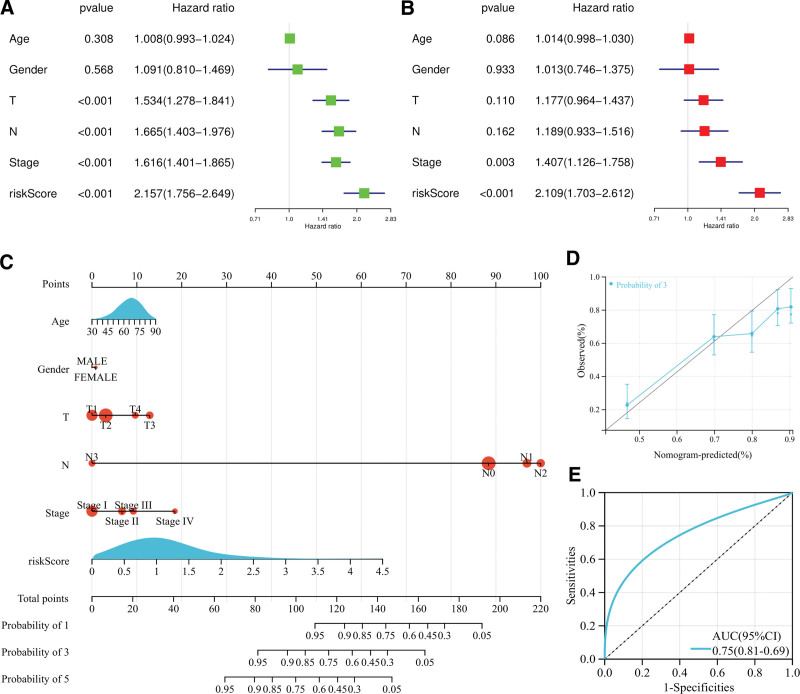
Prediction of prognosis of LUAD patients at risk model. (A) Univariate prognostic analysis. (B) Multivariate independent prognostic analysis. (C) Nomogram of the prognostic model. (D) Calibration charts tested the accuracy of the model in predicting 3- year prognosis. (E) ROC curve tests the accuracy of the model to predict the 3-year survival state.

In order to further evaluate the predictive value of the above risk models, Univariate and multivariate Cox regression analysis were carried out. Univariate analysis showed that the metabolic related gene risk score model could be used as a predictor of the prognosis of LUAD (Fig. 6A). Multivariate analysis showed that the risk score could independently predict the prognosis of LUAD (HR = 2.109, *P < *.001) (Fig. 6B). The “rms” package is used to plot nomograms to predict LUAD patient survival. Clinical factors and risk score were used to predict survival of LUAD (Fig. 6C). The 3-year actual survival rate of the calibration curve coincides well with the predicted survival rate (Fig. 6D). ROC curve shows that the AUC value of 3-year survival rate was 0.75 (Fig. 6E).

### 3.6. Correlation between immune cell infiltration and risk model

**Figure 7.**
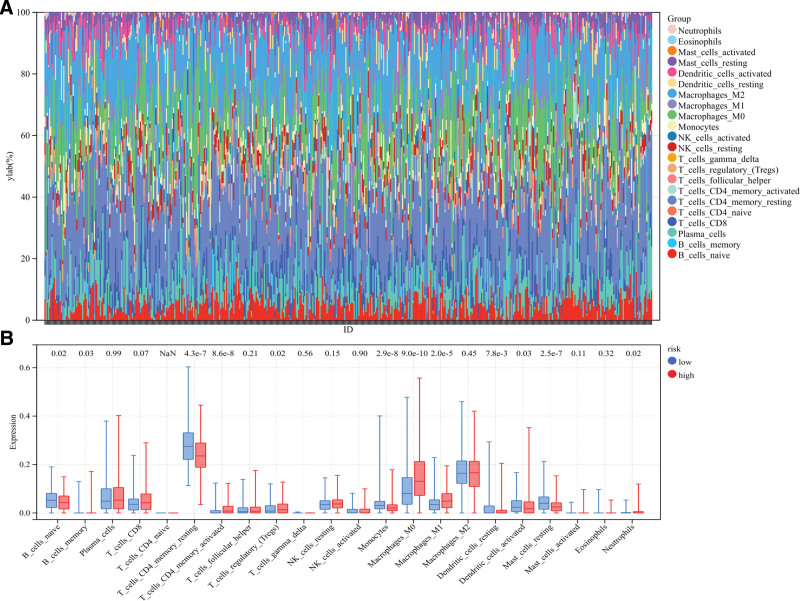
Difference of immune cell infiltration in different risk subgroups. (A) Histogram of the proportion of 22 immune cells in LUAD tissue. Each cell type is represented by a different color. Each column represents an LUAD sample ID. (B) Box plot shows differences in immune cell infiltration between high and low risk groups.

**Figure 8.**
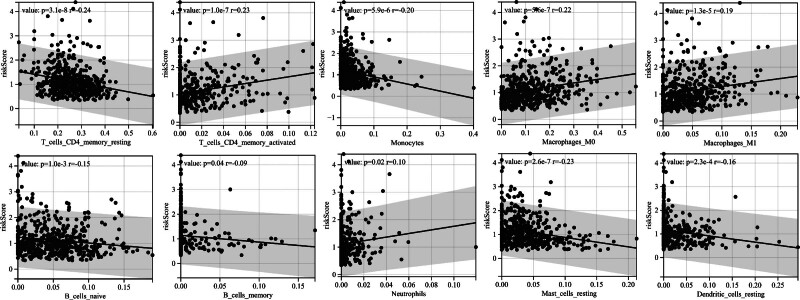
Correlation between risk score and immune cell infiltration level. *R* value stands for correlation coefficient, and positive number stands for positive correlation.

In the study of tumor microenvironment, CIBERSORT method is used to calculate the proportion of immune cell subsets. CIBERSORT method was used to analyze the composition of immune cells in LUAD samples (Fig. 7A). Differential analysis of the infiltration ratios of 22 types of immune cells in patients from different risk groups was performed (Fig. 7B). Results show T cells CD4 memory activated (*P* = 8.6e−8), T cells regulatory (Tregs) (*P* = .02), Macrophages M0 (*P* = 9.0e−10), macrophages M1 (*P* = 2.0e−5) and neutrophils (*P* = .02) were significantly clustered in the high-risk group. B cells naive (*P* = .02), T cells CD4 memory resting (*P* = 4.3e−7), monocytes (*P* = 2.9e−8), dendritic cells resting (*P* = 7.8e−3) and mast cells resting (*P* = 2.5e−7) were clustered in the low-risk subgroup. Pearson correlation analysis showed that the infiltration level of T cells CD4 memory resting (*r* = −0.24, *P* = 3.1e−8), T cells CD4 memory activated (*R* = 0.23, *P* = 1.0e−7), monocyte (*r* = −0.20, *P* = 5.9e−6), Macrophages M0 (*R* = 0.22, *P* = 5.6e−7), macrophages M1 (*R* = 0.19, *P* = 1.3e−5), B cells naive (*r* = −0.15, *P* = 1.0e−3), B cells memory (*r* = −0.09, *P* = .04), neutrophils (*R* = 0.10, *P* = .02), mast cells resting (*r* = −0.23, *P* = 2.6e−7) and dendritic cells resting (*r* = −0.16, *P* = 2.3e−4) were related to the risk score (Fig. 8).

### 3.7. Validation of model genes

**Figure 9.**
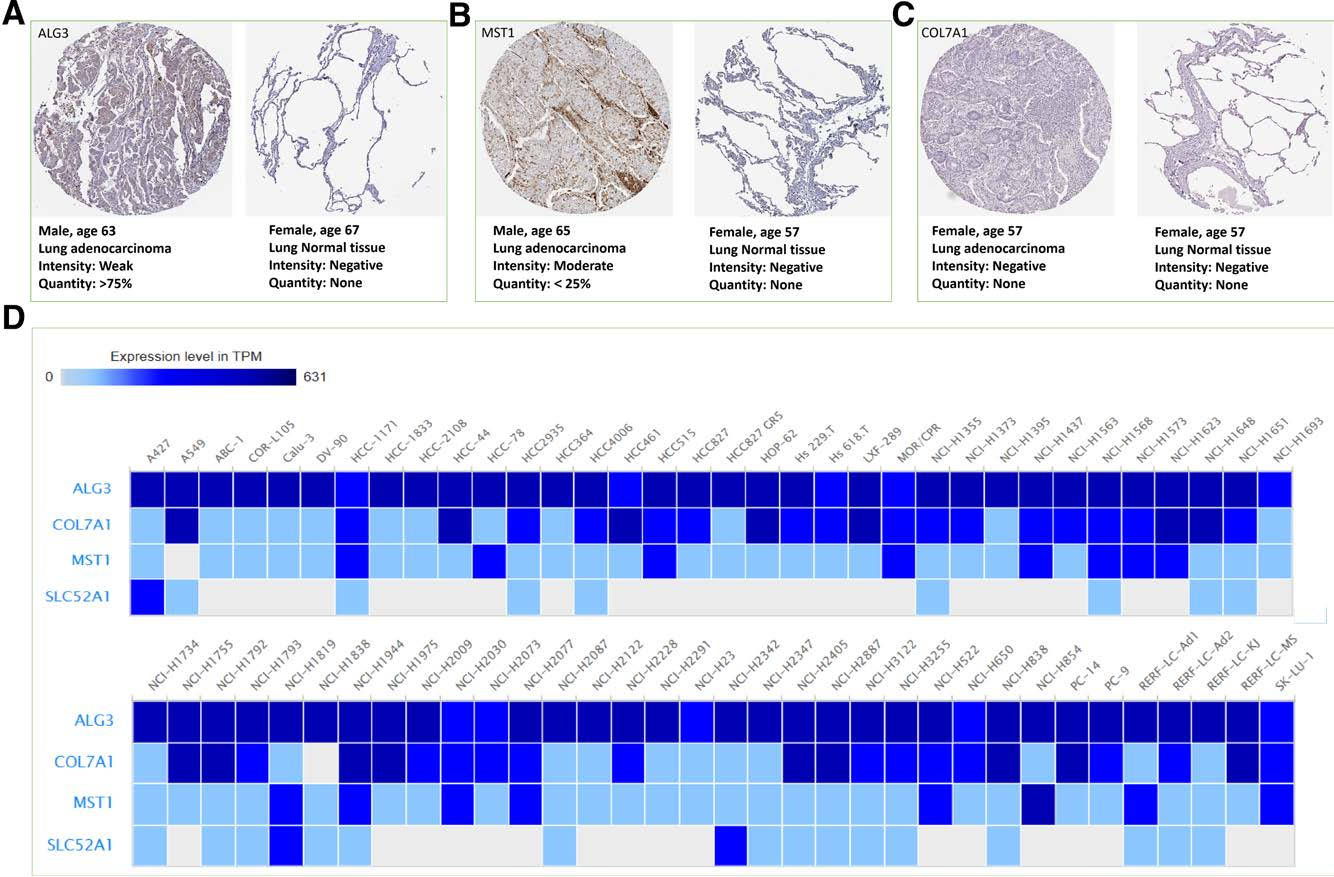
Expression verification of model genes. (A) Expression of ALG3 protein in cancer and normal control tissues. (B) Expression of MST1 protein in cancer and normal control tissues. (C) Expression of COL7A1 protein in cancer and normal control tissues. (D) Expression levels of model genes (ALG3, COL7A1, MST1, and SLC52A1) in LUAD cell lines.

**Figure 10.**
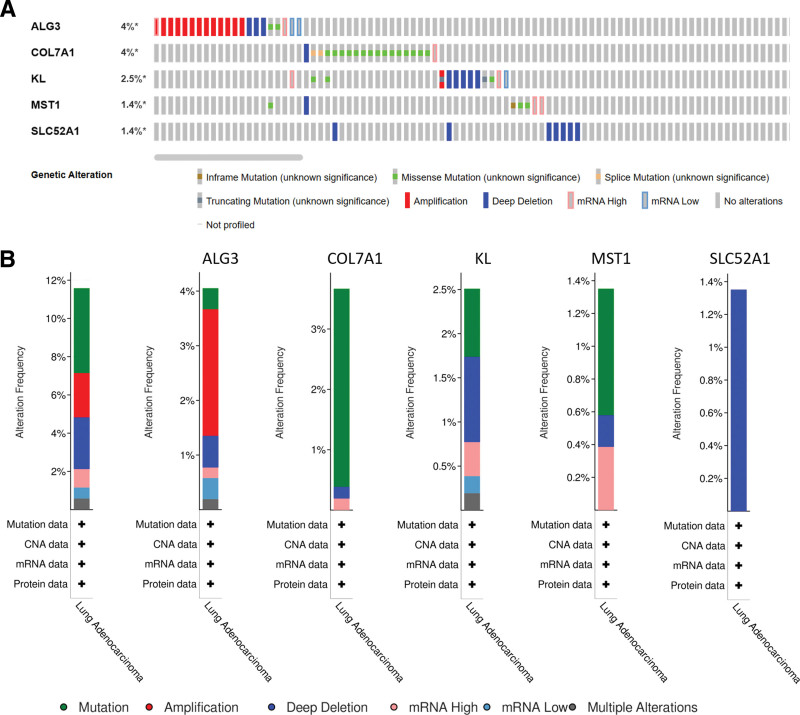
Model gene mutation analysis. (A) Mutation of model genes in clinical cases. (B) Percentage of mutation types in model genes.

In this study, the expression of model genes in normal tissues and lung adenocarcinoma tissues was analyzed according to the immunohistochemical results of histological and pathological maps in HPA database. The results showed that the expressions of ALG3, COL7A1 and SLC52A1 were upregulated in LUAD tissues (Fig. 9A to C). In addition, ALG3 and COL7A1 were highly expressed in most LUAD cell lines (Fig. 9D). In addition, based on LUAD sample data in cBioPortal database, the genetic alteration of 5 model genes was analyzed. Among LUAD patients, the genetic alteration rates of ALG3, COL7A1, KL, MST1, and SLC52A1 were 4%, 4%, 2.5%, 1.4%, and 1.4%, respectively (Fig. 10A). The main types of mutations in model genes include mutation, amplification and deep deletion. Among them, the main mutation type of ALG3 was amplification. The main mutation type that occurs in COL7A1 was mutation. The main types of mutations that occur in KL were mutation and deep deletion. The main types of mutations that occur in MST1 were mutation and mRNA High. The main mutation type of SLC52A1 was deep deletion (Fig. 10B).

### 3.8. Construction of model gene ceRNA network

**Figure 11.**
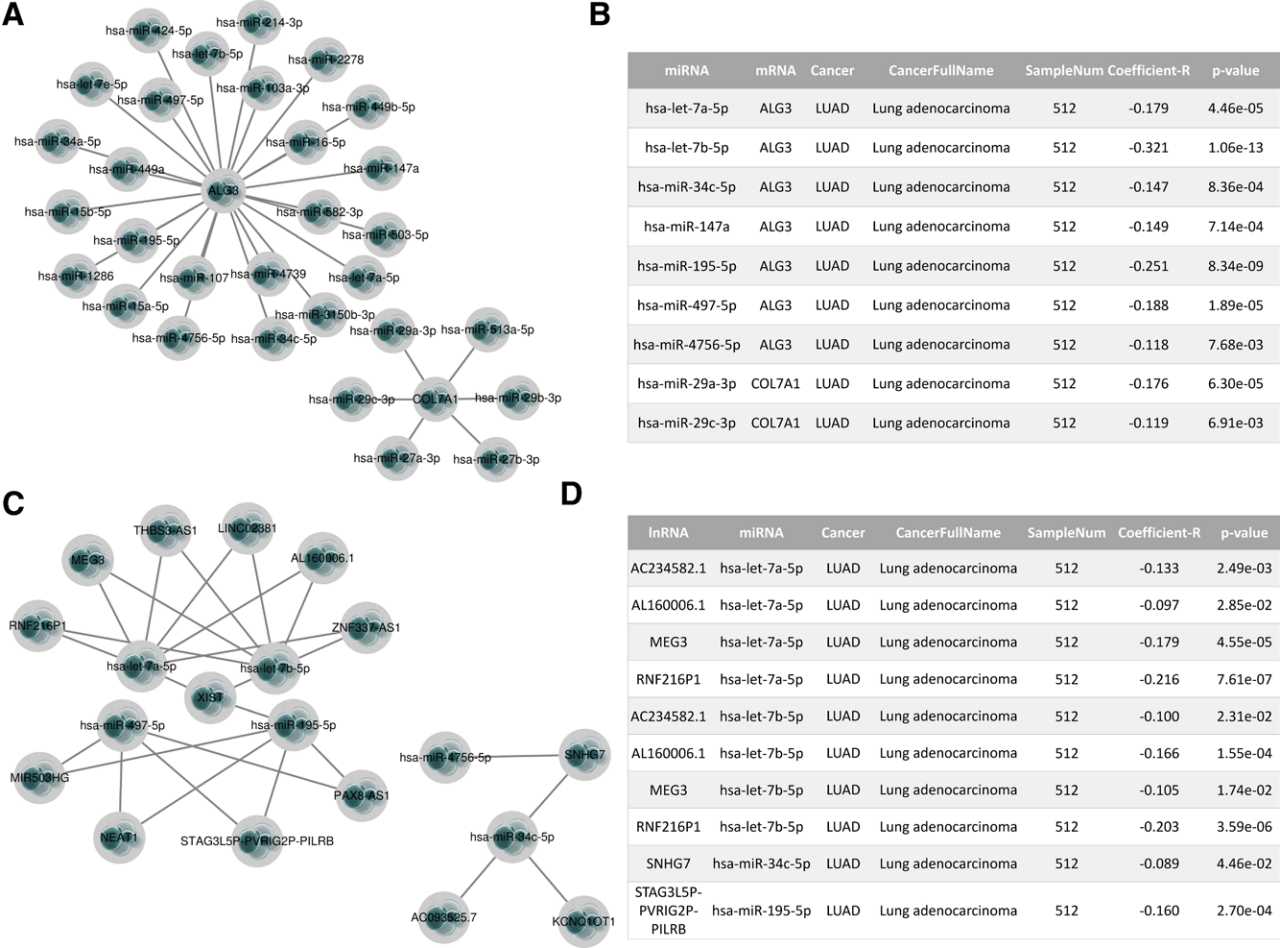
Construction of the “lncRNA-miRNA-mRNA” ceRNA regulatory network of the prediction model gene in the starBase database. (A) Model gene mRNA-miRNA regulatory network. (B) MiRNA negatively correlated with model gene mRNA in LUAD. (C) LncRNA-miRNA regulatory network. (D) LncRNA negatively associated with miRNA in LUAD.

**Figure 12.**
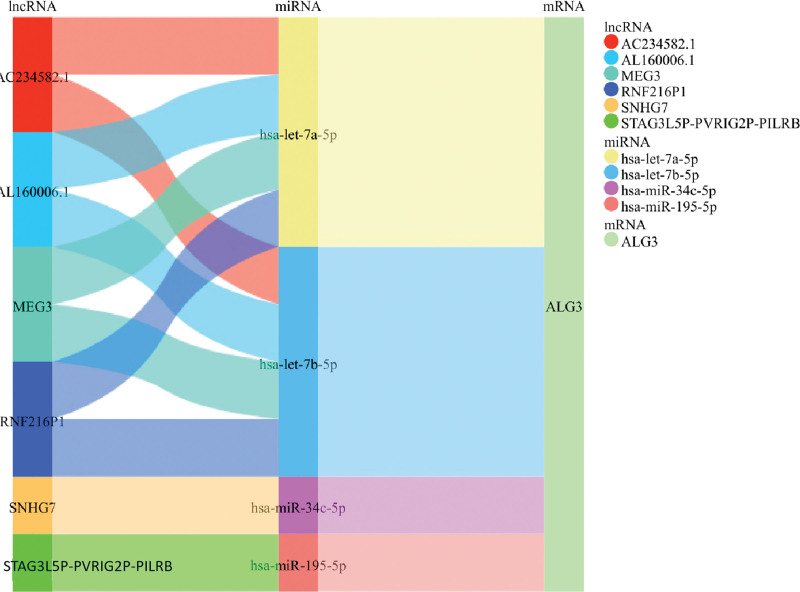
Construction of lncRNA-miRNA-mRNA regulatory network by mulberry map.

More and more evidence shows that the lncRNA-miRNA-mRNA regulatory mechanism exists widely in cancer. Therefore, we tried to explore the mechanism of ceRNA regulation of model genes. We predicted the target miRNAs of the model mRNA, 30 of which may be bound to the model gene mRNA (Fig. 11A). In addition, we predicted the correlation between mRNA and miRNA. Correlation analysis showed that the expression of 9 upstream miRNAs was negatively correlated with the model mRNA, namely hsa-let-7a-5p, hsa-let-7b-5p, hsa-miR-34c-5p, hsa-miR-147a, hsa-miR-195-5p, hsa-miR-497-5p, hsa-miR-4756-5p, hsa-miR-29a-3p, hsa-miR-29c-3p (Fig. 11B). Subsequently, we further predicted upstream lncRNAs of the above miRNAs (Fig. 11C). In the correlation analysis between miRNAs and lncRNAs, the expression of AC234582.1, AL160006.1, MEG3, and RNF216P1 was negatively correlated with hsa-let-7a-5p. The expression of AC234582.1, AL160006.1, MEG3, and RNF216P1 was negatively correlated with hsa-let-7b-5p. The expression of SNHG7 was negatively correlated with hsa-miR-34c-5p. The expression of STAG3L5P-PVRIG2P-PILRB was negatively correlated with hsa-miR-195-5p (Fig. 11D). Based on the above analysis results, we constructed the ceRNA network of model genes (Fig. 12).

### 3.9. Expression of ALG3 in LUAD

**Figure 13.**
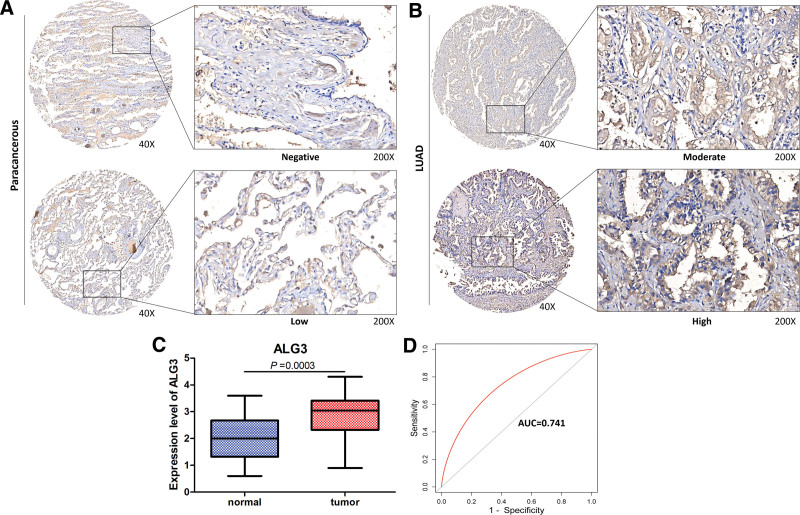
Expression validation of ALG3 in LUAD. (A) Expression of ALG3 protein in paracancerous tissues. (B) Expression of ALG3 protein in LUAD tissues. (C) Differential expression analysis of ALG3 in cancer and paracancerous tissues in tissue expression microarray. Paired sample t-test was used to compare cancer and paracancerous samples. (D) The ROC curve was used to analyze the diagnostic effect of ALG3 on LUAD.

Expression of the key model gene ALG3 was validated in LUAD tissues. Tissue microarrays constructed using 31 LUAD and 28 paraneoplastic samples were used and immunohistochemistry was used to validate ALG3 expression in cancer and neighboring cancers. ALG3 was strongly expressed in cancer tissues compared to paraneoplastic tissues (Fig. 13A to B). Paired *t*-test was used to detect differences in ALG3 expression between cancer and neighboring samples, and ALG3 expression was found to be upregulated in cancer (Fig. 13C).The ROC curve showed that ALG3 had a good diagnostic efficiency for cancer (AUC = 0.741) (Fig. 13D).

## 4. Discussion

Many changes have taken place in tumor cells to meet their energy and material needs, and metabolic changes have become an important feature. More and more people pay attention to the study of metabolic related genes to judge the characteristics and prognosis of tumors.^[9]^ For example, the prognosis model of lung adenocarcinoma based on immuno-glycolysis-related genes has a good prediction effect on the survival of patients.^[10]^ Therefore, in this study, abnormal metabolism related genes were used to judge the prognosis of LUAD. In this study, mRNA expression profile data of LUAD patients and control samples were downloaded from TCGA database. Genes related to abnormal metabolism were extracted for further difference analysis. Finally, 42 differential genes related to abnormal metabolism were screened. Five genes related to prognosis were identified. The 5 genes used to construct the prognosis model include ALG3, COL7A1, KL, MST1, and SLC52A1.

Previous studies have shown that the above model genes have great predictive ability for the survival of lung adenocarcinoma and other tumors. ALG3 gene encodes a member of the ALG3 (alpha-1,3-mannosyltransferase) family and is associated with glycosylation diseases and protein metabolism. It has been demonstrated that ALG3 is upregulated in NSCLC tissues and cells, and patients with high ALG3 expression have a poorer prognosis. Overexpression of ALG3 promotes the malignant phenotype and EMT process in NSCLC cells.^[11]^ Consistent with previous results, ALG3 expression was upregulated in LUAD tissues and had good diagnostic efficacy for LUAD. Type VII collagen (encoded by the COL7A1 gene) acts as an anchoring protofiber for the basement membrane and contributes to epithelial basement membrane organization and adhesion by interacting with extracellular matrix (ECM) proteins such as type IV collagen. COL7A1 was more densely expressed in gastric cancer tissues and malignant esophageal cancer tissues than in normal tissues, and high COL7A1 expression was associated with tumor infiltration, metastasis, and poor patient prognosis.^[12]^ Klotho (KL) is a classical senescence suppressor gene. It has been found that Klotho gene polymorphisms are associated with tumor development and growth. As a valuable tumor suppressor gene, the KL protein is expressed at a low rate in various cancerous tissues, including renal, breast, liver, lung, and pancreatic cancers, which elucidates its ability to inhibit tumor cell growth.^[13]^ Mammalian STE20-like kinase 1 (MST1) is a Hepatocyte growth factor-like protein alpha chain and a link in the Hippo pathway. Studies have shown that MST1 exerts tumor suppressor effects by regulating apoptosis, migration and proliferation of colorectal and lung cancer cells.^[14]^ Solute carrier family 52 member 1 (SLC52A1) plays a critical role in the biochemical redox reactions of carbohydrate, lipid, and amino acid metabolism. Deficiency of SLC52A1 directly enhances immunosuppressive activity by facilitating STAT3-mediated reactive oxygen species production, contributing to unfavorable prognostic factors in cancer.^[15]^ Among the above genes, ALG3, COL7A1, MST1, and SLC52A1 were upregulated in LUAD and KL was downregulated in LUAD, and all of them were associated with patient prognosis. ALG3 and COL7A1 were potential risk factors for LUAD and may be potential targets for antitumor therapy. Although the above genes are closely related to the prognosis of LUAD, this study is the first time to use them in combination to predict the survival rate of LUAD.

In this study, 5 prognostic related genes (ALG3, COL7A1, KL, MST1, and SLC52A1) were used to establish a LUAD polygenic prognostic risk model. The distribution of survival status indicates that the death population is more dense in the high-risk population. The prognosis of LUAD patients in the high-risk subgroup is poor. This model has great prediction efficiency for LUAD. The prediction efficiency of the LUAD risk model was also verified in the GSE50081 data set. Cox regression analysis was conducted to further evaluate the predictive value of the above risk model. Multivariate analysis showed that the model score could independently predict the prognosis of LUAD. The above studies show that the risk model constructed by the combination of 5 genes can better predict the prognosis of LUAD patients.

It is well known that immune infiltration is associated with tumor progression, and its function in the development of malignant tumors has been confirmed.^[16,17]^ In our study, we used the CIBERSORT method to calculate the immune cell infiltration in LUAD patients, and analyzed the relationship with the risk model. Results show T-cells CD4 memory activated, T cells regulatory (Tregs), macrophages M0, macrophages M1 and neutrophils were significantly clustered in the high-risk group. B cells naive, T cells CD4 memory resting, monocytes, dendritic cells resting and mast cells resting were clustered in the low-risk subgroup. B cell infiltration is a marker of improved prognosis in lung cancer, and the lower the tumor grade, the higher the proportion of naive B cells.^[18]^ T cells regulatory (Tregs) can inhibit the function of CD8 T cells through self-secreted cytokines and promote tumor progression to a certain extent.^[19]^ The degree of CD4 + T cell memory activation correlated with the malignancy of the tumor.^[20]^ The higher degree of CD4 T-cell memory activation in the high-risk group and the higher degree of CD4T-cell memory quiescence in the low-risk group indicated that the degree of malignancy of LUAD tumors was higher in the high-risk group, which indirectly indicated that the prediction model had a higher risk prediction ability for LUAD. It has been shown that CD68 + HLA-DR + M1-type macrophage enhances tumor cell motility in hepatocellular carcinoma.^[21]^ Exosomes in oral squamous cell carcinoma regulate the conversion of macrophage to M1, which promotes malignant tumor metastasis.^[22]^ The pro-tumorigenic effects of M1 macrophages may be due to inflammatory cytokines. Increased levels of M0-type and M1-type macrophage infiltration predicted high malignancy of LUAD in the high-risk group. In the immune microenvironment, neutrophils can produce a variety of factors that promote tumor development.^[23]^ For example, they can produce growth factors and angiogenic factors to help tumors grow and spread. In some studies, an increase in the number of neutrophils in the tissues of patients with tumors has been associated with a poor prognosis.^[24]^ Increased levels of neutrophil infiltration in the high-risk group were consistent with the risk model’s prediction of a poor prognosis for patients with LUAD. Similarly, T cells CD4 memory activated, Macrophages M0, Macrophages M1 and Neutrophils were positively correlated with the risk score of LUAD patients. T cells CD4 memory resting, B cells naive, mast cells resting, dendritic cells resting and Monocyte were negatively correlated with the risk score of LUAD patients. Results of immune infiltration explain the potential mechanism by which LUAD risk model predicts patient prognosis. Inhibiting the regulatory work of cancer promoting immune cells may become a potential therapeutic strategy.

The crosstalk between ceRNAs is realized by the competitive combination of miRNA and long noncoding RNA (lncRNA), so as to further regulate the expression of mRNA.^[25]^ LncRNA acts as a miRNA sponge to reduce the content of miRNA in cells, thus interfering with the inhibition of downstream target genes by miRNA.^[26,27]^ In our study, we predicted upstream miRNAs of model genes (ALG3, COL7A1, KL, MST1, and SLC52A1). Based on the ceRNA network hypothesis, 9 regulatory mechanisms (hsa-let-7a-5p, hsa-let-7b-5p, hsa-miR-34c-5p, hsa-miR-147a, hsa-miR-195-5p, hsa-miR-497-5p, hsa-miR-4756-5p, hsa-miR-29a-3p and hsa-miR-29c-3p) were found. Finally, we further predicted regulatory lncRNA upstream of miRNA. Through correlation analysis, the lncRNA bound upstream of the miRNA-mRNA axis was screened (Fig. 11D), and the mulberry map was drawn to fully understand the potential ceRNA regulatory network of LUAD (Fig. 12).

In conclusion, metabolism-related genes obtained by screening can significantly affect the metabolism of LUAD patients, which in turn affects their prognosis. Established prognostic models can help clinicians provide useful references for patients’ prognosis, and risk scores are associated with multiple immune cell infiltrations. IHC experiments revealed that the metabolic abnormality-associated gene, ALG3, may be a potential diagnostic and prognostic biomarker for LUAD. However, there are some limitations of this study. Prognostic models need to be further validated in prospective cohorts with larger sample sizes. The ceRNA regulatory network proposed in this study provides a theoretical framework for understanding molecular interactions, but experimental validation is needed to confirm the functional relevance of these interactions in the pathogenesis of LUAD.

## Acknowledgments

We would like to thank the team members for their contributions to this paper, and then we will continue to work hard to do relevant research.

## Author contributions

**Conceptualization:** Xinying Wang.

**Data curation:** Abdusemer Reyimu, Aihemaitijiang Kaisaier, Yinzhong Sha, Pawuziye Paerhati, Maimaituxun Maimaiti.

**Formal analysis:** Aihemaitijiang Kaisaier.

**Funding acquisition:** Xiang Cheng, Wen Liu.

**Methodology:** Abdusemer Reyimu, Xiang Cheng, Aihemaitijiang Kaisaier, Yinzhong Sha, Ruijie Guo, Pawuziye Paerhati, Maimaituxun Maimaiti.

**Project administration:** Chuanjiang He, Li Li, Xiaoguang Zou, Aimin Xu.

**Supervision:** Wen Liu, Xinying Wang, Pawuziye Paerhati, Chuanjiang He, Li Li, Xiaoguang Zou, Aimin Xu.

**Validation:** Wen Liu, Xinying Wang, Ruijie Guo, Chuanjiang He, Xiaoguang Zou, Aimin Xu.

**Visualization:** Yinzhong Sha.

**Writing – original draft:** Abdusemer Reyimu, Xiang Cheng.

## Supplementary Material



## References

[1] Abu RF Singhi EK Sridhar A Faisal MS Desai A. Lung cancer treatment advances in 2022. Cancer Invest. 2023;41:12–24. 36036470 10.1080/07357907.2022.2119479

[2] Leiter A Veluswamy RR Wisnivesky JP. The global burden of lung cancer: current status and future trends. Nat Rev Clin Oncol. 2023;20:624–39. 37479810 10.1038/s41571-023-00798-3

[3] Wang S Lv J Lv J. Prognostic value of lactate dehydrogenase in non-small cell lung cancer patients with brain metastases: a retrospective cohort study. J Thorac Dis. 2022;14:4468–81. 36524070 10.21037/jtd-22-1502 PMC9745527

[4] Lin X Xiao Z Chen T Liang SH Guo H. Glucose metabolism on tumor plasticity, diagnosis, and treatment. Front Oncol. 2020;10:317. 32211335 10.3389/fonc.2020.00317 PMC7069415

[5] Xia L Oyang L Lin J. The cancer metabolic reprogramming and immune response. Mol Cancer. 2021;20:28. 33546704 10.1186/s12943-021-01316-8 PMC7863491

[6] Zhao L Mao Y Zhao Y Cao Y Chen X. Role of multifaceted regulators in cancer glucose metabolism and their clinical significance. Oncotarget. 2016;7:31572–85. 26934324 10.18632/oncotarget.7765 PMC5058779

[7] Robinson MD McCarthy DJ Smyth GK. edgeR: a bioconductor package for differential expression analysis of digital gene expression data. Bioinformatics. 2010;26:139–40. 19910308 10.1093/bioinformatics/btp616 PMC2796818

[8] Yu G Wang LG Han Y He QY. clusterProfiler: an R package for comparing biological themes among gene clusters. Omics. 2012;16:284–7. 22455463 10.1089/omi.2011.0118 PMC3339379

[9] Reznik E Sander C. Extensive decoupling of metabolic genes in cancer. PLoS Comput Biol. 2015;11:e1004176. 25961905 10.1371/journal.pcbi.1004176 PMC4427321

[10] Zhang Y Qin W Zhang W Qin Y Zhou YL. Guidelines on lung adenocarcinoma prognosis based on immuno-glycolysis-related genes. Clin Transl Oncol. 2023;25:959–75. 36447119 10.1007/s12094-022-03000-9 PMC10025218

[11] Ke SB Qiu H Chen JM. ALG3 contributes to the malignancy of non-small cell lung cancer and is negatively regulated by MiR-98-5p. Pathol Res Pract. 2020;216:152761. 31899049 10.1016/j.prp.2019.152761

[12] Oh SE Oh MY An JY. Prognostic value of highly expressed type VII collagen (COL7A1) in patients with gastric cancer. Pathol Oncol Res. 2021;27:1609860. 34512204 10.3389/pore.2021.1609860 PMC8426344

[13] Pan KH Yao L Chen Z. KL is a favorable prognostic factor related immune for clear cell renal cell carcinoma. Eur J Med Res. 2023;28:356. 37726833 10.1186/s40001-023-01242-z PMC10510209

[14] Zhang W Liu K Pei Y Ma J Tan J Zhao J. Mst1 regulates non-small cell lung cancer A549 cell apoptosis by inducing mitochondrial damage via ROCK1/F-actin pathways. Int J Oncol. 2018;53:2409–22. 30320378 10.3892/ijo.2018.4586 PMC6203146

[15] Zhang X Shi X Zhao H Jia X Yang Y. Identification and validation of a tumor microenvironment-related gene signature for prognostic prediction in advanced-stage non-small-cell lung cancer. Biomed Res Int. 2021;2021:8864436. 33860055 10.1155/2021/8864436 PMC8028741

[16] Lin B Du L Li H Zhu X Cui L Li X. Tumor-infiltrating lymphocytes: warriors fight against tumors powerfully. Biomed Pharmacother. 2020;132:110873. 33068926 10.1016/j.biopha.2020.110873

[17] Thomopoulou K Papadaki C Monastirioti A. MicroRNAs regulating tumor immune response in the prediction of the outcome in patients with breast cancer. Front Mol Biosci. 2021;8:668534. 34179081 10.3389/fmolb.2021.668534 PMC8220200

[18] Chen J Tan Y Sun F. Single-cell transcriptome and antigen-immunoglobin analysis reveals the diversity of B cells in non-small cell lung cancer. Genome Biol. 2020;21:152. 32580738 10.1186/s13059-020-02064-6 PMC7315523

[19] Zhou X Zhao S He Y Geng S Shi Y Wang B. Precise spatiotemporal interruption of regulatory T-cell-mediated CD8(+) T-cell suppression leads to tumor immunity. Cancer Res. 2019;79:585–97. 30254146 10.1158/0008-5472.CAN-18-1250

[20] Li M Zhao J Yang R. CENPF as an independent prognostic and metastasis biomarker corresponding to CD4+ memory T cells in cutaneous melanoma. Cancer Sci. 2022;113:1220–34. 35189004 10.1111/cas.15303 PMC8990861

[21] Wang H Wang X Li X. CD68(+)HLA-DR(+) M1-like macrophages promote motility of HCC cells via NF-κB/FAK pathway. Cancer Lett. 2014;345:91–9. 24333724 10.1016/j.canlet.2013.11.013

[22] Xiao M Zhang J Chen W Chen W. M1-like tumor-associated macrophages activated by exosome-transferred THBS1 promote malignant migration in oral squamous cell carcinoma. J Exp Clin Cancer Res. 2018;37:143. 29986759 10.1186/s13046-018-0815-2 PMC6038304

[23] Hajizadeh F Aghebati ML Alexander M. Tumor-associated neutrophils as new players in immunosuppressive process of the tumor microenvironment in breast cancer. Life Sci. 2021;264:118699. 33137368 10.1016/j.lfs.2020.118699

[24] Cupp MA Cariolou M Tzoulaki I Aune D Evangelou E Berlanga-Taylor AJ. Neutrophil to lymphocyte ratio and cancer prognosis: an umbrella review of systematic reviews and meta-analyses of observational studies. BMC Med. 2020;18:360. 33213430 10.1186/s12916-020-01817-1 PMC7678319

[25] Zhang K Zhang L Mi Y. A ceRNA network and a potential regulatory axis in gastric cancer with different degrees of immune cell infiltration. Cancer Sci. 2020;111:4041–50. 32860283 10.1111/cas.14634 PMC7648034

[26] Chan JJ Tay Y. Noncoding RNA:RNA regulatory networks in cancer. Int J Mol Sci . 2018;19:1310. 29702599 10.3390/ijms19051310 PMC5983611

[27] Swain AC Mallick B. miRNA-mediated “tug-of-war” model reveals ceRNA propensity of genes in cancers. Mol Oncol. 2018;12:855–68. 29603582 10.1002/1878-0261.12198 PMC5983123

